# Comparison of the gut microbiota composition between obese and non-obese individuals in a Japanese population, as analyzed by terminal restriction fragment length polymorphism and next-generation sequencing

**DOI:** 10.1186/s12876-015-0330-2

**Published:** 2015-08-11

**Authors:** Chika Kasai, Kazushi Sugimoto, Isao Moritani, Junichiro Tanaka, Yumi Oya, Hidekazu Inoue, Masahiko Tameda, Katsuya Shiraki, Masaaki Ito, Yoshiyuki Takei, Kojiro Takase

**Affiliations:** 1 Department of Gastroenterology, Mie Prefectural General Medical Center, 5450-132 Hinaga, Yokkaichi, Mie 510-8561 Japan; 2 Department of Molecular and Laboratory Medicine, Mie University School of Medicine, 2-174 Edobashi, Tsu, Mie 514-8507 Japan; 3 Department of Gastroenterology and Hepatology, Mie University School of Medicine, Tsu, Japan; 4 Department of Cardiology and Nephrology, Tsu, Japan

**Keywords:** Obesity, Microbiota, T-RFLP, Next-generation sequencing

## Abstract

**Background:**

Obesity has become one of the most serious social problems in developed countries, including Japan. The relationship between the gut microbiota and obesity has recently attracted the attention of many researchers. Although the gut microbiota was long thought to contribute to obesity, the exact association remains largely unknown. We examined the human gut microbiota composition in a Japanese population in order to determine its relationship to obesity.

**Methods:**

Stool samples from 23 non-obese subjects (body mass index [BMI] <20 kg/m^2^) and 33 obese subjects (BMI ≥25 kg/m^2^) were collected and DNA was extracted prior to colonoscopy. After terminal restriction fragment length polymorphism (T-RFLP) analysis, samples from 10 subjects (4 non-obese and 6 obese) were selected and subjected to next-generation sequencing for species-level analysis.

**Results:**

T-RFLP analysis showed significantly reduced numbers of *Bacteroidetes* and a higher *Firmicutes* to *Bacteroidetes* ratio in obese subjects compared with non-obese subjects. Bacterial diversity was significantly greater in obese subjects compared with non-obese subjects. Next-generation sequencing revealed that obese and non-obese subjects had different gut microbiota compositions and that certain bacterial species were significantly associated with each group (obese: *Blautia hydrogenotorophica*, *Coprococcus catus*, *Eubacterium ventriosum*, *Ruminococcus bromii*, *Ruminococcus obeum*; non-obese: *Bacteroides faecichinchillae*, *Bacteroides thetaiotaomicron*, *Blautia wexlerae*, *Clostridium bolteae*, *Flavonifractor plautii*).

**Conclusion:**

Gut microbial properties differ between obese and non-obese subjects in Japan, suggesting that gut microbiota composition is related to obesity.

**Electronic supplementary material:**

The online version of this article (doi:10.1186/s12876-015-0330-2) contains supplementary material, which is available to authorized users.

## Background

With the increasing westernization of Japanese dietary habits, obesity has become a serious social problem that is associated with metabolic disorders, including such lifestyle-associated diseases as diabetes. Numerous recent studies have revealed that the human gut microbiota is strongly associated with host energy regulation and homeostasis, thereby affecting the clinical conditions of diabetic and/or obese patients [1].

The human gut is continually colonized by complex microbial communities in which the combined number of cells (10^11-13^ cells per gram range in the colon) is greater than the total number of host cells [2]. That is to say, the human body harbors 10 times as many exogenous cells as its own. Recently, the gut microbiota was referred to as a “super organism” [3] and a “virtual organ” [4] because it affects both the biology and physiology of the host. However, numerous details regarding the precise mechanism underlying the effects of gut microbiota activities on host homeostasis remain to be elucidated. Recent research has revealed that the composition of the gut microbiota varies with age, dietary habits, geographic environment, and other host-associated factors [5–8].

As the human gut microbiota is comprised primarily of anaerobes, 60-80 % of which are uncultivatable [9], traditional culture methods are of limited usefulness for studying these organisms. However, owing to recent developments in molecular biological methods and the increasing utility of next-generation sequencing technology (both of which allow the detection of uncultivatable microbiomes), this research area has shown a notable advance.

Using one such molecular biological method, 16S rRNA sequencing, Turnbaugh et al. employed a genomic approach to examine the role of the gut microbiota (traditionally recognized as being associated with energy harvesting in the host) in the development of obesity [10, 11]. Through animal studies involving ob/ob mice, they found that obesity is associated with changes in the relative abundance of two dominant bacterial phyla, the *Bacteroidetes* and the *Firmicutes*. The ob/ob mice harbored fewer *Bacteroidetes* and more *Firmicutes* than did lean mice [11]. Moreover, a human study showed that the microbiota of obese subjects is less diverse and is composed of significantly fewer *Bacteroidetes* compared with non-obese subjects [12]. Similar results were observed in another study by Armougom et al., who reported a significant reduction in the proportion of *Bacteroidetes* in obese patients compared with lean individuals [13]. However, other research has contradicted these findings. A significant increase in the proportion of *Bacteroides* in obese and overweight subjects compared with lean controls has been reported [14], whereas other researchers have found no correlation between human obesity and the proportions of *Bacteroides* and *Firmicutes* among fecal bacteria [15].

Thus, previous research suggests that the composition of the gut microbiota differs between obese and non-obese subjects. However, the results of studies in humans and mice have been inconsistent, generating considerable controversy as to the proportions of *Bacteroides* and *Firmicutes* and their relationship to obesity. Primarily led by Western researchers, considerable attention has focused on studies of the relationship between the gut microbiota and various diseases. However, to the best of our knowledge, only a limited number of studies have addressed this topic in Japanese populations, whose dietary habits differ from Western populations. Furthermore, previous research done in Japan has not adequately analyzed the relationship between the gut microbiota at the species level and disease development. Therefore, in this study, we investigated the human gut microbiota in a Japanese population using next-generation sequencing in addition to terminal restriction fragment length polymorphism (T-RFLP) analysis which has been revealed to be useful for analyzing gut microbiota [16]. We identified 10 potential bacterial species uniquely associated with obesity and non-obesity.

## Methods

### Human subjects

Subjects who were under 65 years of age and had undergone colonoscopy at Mie Prefectural General Medical Center, Yokkaichi, Japan, between 2012 and 2013 were enrolled in the study.

According to the definition of the Japan Society for the Study of Obesity, subjects with a body mass index (BMI) <18.5 kg/m^2^ are classified as lean, whereas subjects with a BMI between 18.5 and 25 kg/m^2^ are classified as normal, and those with a BMI ≥25 kg/m^2^ were classified as obese. In the current study, we classified subjects with a BMI <20 kg/m^2^ as non-obese, and those with a BMI ≥25 kg/m^2^ as obese, for we did not have enough subjects with a BMI <18.5 kg/m^2^ to make statistical analyses.

**Fig. 1:**
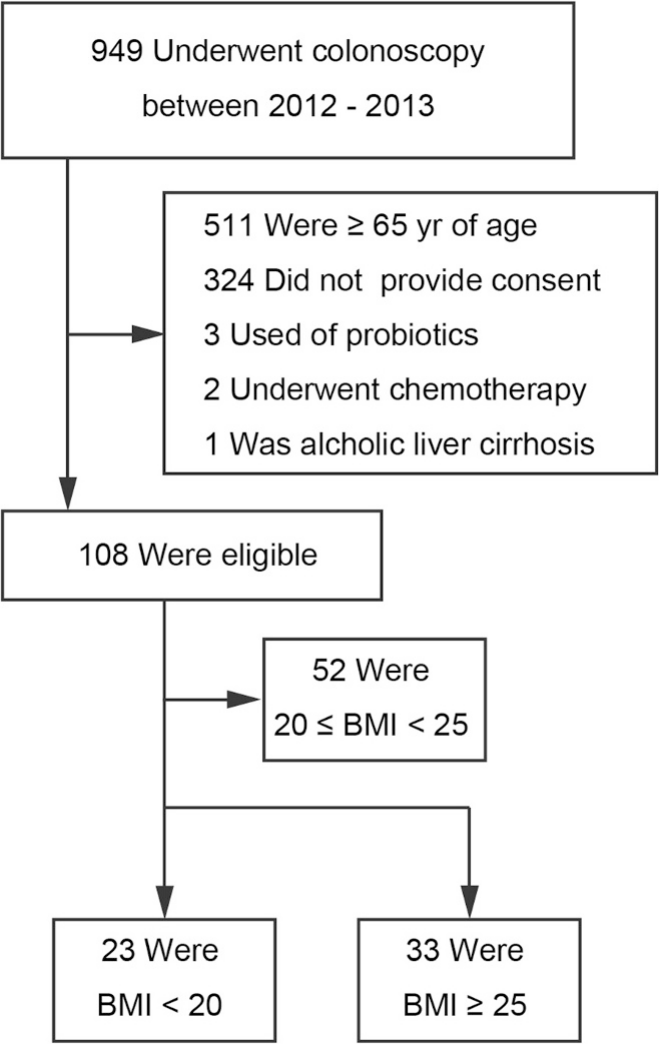
Flowchart showing the total number of participants enrolled and the final number of participants included in the study. 23 with BMI < 20 and 33 with BMI ≥25 were enrolled in the study

Differences in gut microflora between the two groups were evaluated using T-RFLP analysis. Exclusion criteria for all participants included current use of antibiotics, history of or current chronic bowel or liver disease, advanced colorectal cancer, history of chemotherapy or radiation therapy, and regular use of immunosuppressants (steroids, interferon, etc.) or probiotics. Assignment of the patients is shown in Fig. 1. All patients received an explanation of the procedures and possible risks associated with the study and gave written informed consent to participate. This study was performed in conformity with the Declaration of Helsinki and was approved by our institutional ethnics committee (authorization number 2011-5, Mie Prefectural General Medical Center, Yokkaichi, Japan). Stool samples were collected prior to polyethylene glycol preparation of the bowel for colonoscopy. Fecal samples were stored at 4 °C after collection and were submitted to Technosuruga Laboratory (Shizuoka, Japan) for T-RFLP analysis, as described below.

### DNA Extraction

Fecal samples (approximately 4 mg) were immediately suspended in a solution containing100 mM Tris-HCI, pH 9.0, 40 mM Tris-EDTA, pH8.0, and 4 M guanidine thiocyanate, and kept at -20 °C until DNA extraction. An aliquot of 0.8 ml of the suspension was homogenized with zirconia beads in a 2.0 ml screw cap tube by FastPrep 24 Instrument (MP Biomedicals, Santa Ana, CA) at 5 m/s for 2 min and placed on ice for 5 min. After centrifugation at 5000 × g for 1 min, DNA was extracted from 200 μL of the suspension using an automatic nucleic acid extractor (Precision System Science, Chiba, Japan). MagDEA DNA 200 (GC) (Precision System Science) was used as the reagent for automatic nucleic acid extraction.

### T-RFLP

The 16S rDNA was amplified from human fecal DNA using the fluorescent-labeled 516f primer (5’-TGCCAGCAGCCGCGGTA-3’; *Escherichia coli* positions 516 to 532) and 1510r primer (5’-GGTTACCTTGTTACGACTT-3’; *E. coli* positions 1510 to 1492). For this, the Hot-starTaq DNA polymerase by Gene Amp PCR system 9600 (Applied Biosystems, Foster City, CA) was used. The amplification program was as follows: preheating at 95 °C for 15 min, 30 cycles of denaturation at 95 °C for 30 s, annealing at 50 °C for 30 s, extension at 72 °C for 1 min, and finally, a terminal extension at 72 °C for 10 min. The amplified DNA was purified by a MultiScreen PCR96 Filter Plate (Millipore, Billerica, MA) and was verified by electrophoresis. The restriction enzymes were selected according to Nagashima et al. [17, 18]. In brief, the PCR product was purified, and digested with 10 U of *Bsl*I (New England BioLabs, Ipswich, MA) at 55 °C for 3 h. The resultant DNA fragments, namely, fluorescent-labeled terminal restriction fragments (T-RFs), were analyzed by ABIPRISM 3130xl genetic analyzer, and their length and peak area were determined using the genotype software GeneMapper (Applied Biosystems). The T-RFs were divided into 29 operational taxonomic units (OTUs). The OTUs were quantified as the percentage of individual OTU per total OTU areas, which were expressed as the percentage of the area under the curve (%AUC). The bacteria were predicted for each classification unit and the corresponding OTU was identified according to reference Human Fecal Microbiota T-RFLP profiling (http://www.tecsrg-lab.jp/).

From all of the subjects who were diagnosed as normal after colonoscopy (21 of 56), samples from the 4 non-obese subjects with the lowest BMI and the 6 obese subjects with the highest BMI were selected for next-generation sequencing. The diagnosis of other 35 participants are shown in Additional file 1: Table S1.

### Illumina library generation

NGS analysis of microbial community structure in feces was performed using a MiSeq (Illumina, San Diego, CA), as previously described by Takahashi et al. [19]. The V3-V4 region of 16S rDNA was amplified using 341 F (5’-CCTACGGGAGGCAGCAG-3’) [20] and 806R (5’-GGACTACHVGGGTWTCTAAT-3’) [21]. In addition to the V3-V4 specific priming regions, these primers were complementary to standard Illumina forward and reverse primers. The reverse primer also contained a 6-bp indexing sequence (CAGATC, ACTTGA, GATCAG, TAGCTT, GGCTAC, CTTGTA, ATCACG, CGATGT, TTAGGC and TGACCA) to allow for multiplexing. The touchdown PCR method for thermal cycling was used with a GeneAmp PCR system 9700 (ABI, Foster City, CA). The PCR reaction mixture (25 μL) contained 20 ng genomic DNA, 2 × MightyAmp Buffer Ver.2 (Takara, Otsu, Japan), 0.25 μM of each primer, and 1.25 units of MightyAmp DNA Polymerase (Takara). The PCR reaction and preparation of amplicon pool were performed by the method of Takahashi et al. [19].

### Illumina sequencing and quality filtering

Each multiplexed library pool was spiked with 30 % phiX control to improve base calling during sequencing, as recommended by Illumina for the pooling of two libraries, according to Takahashi et al. [19]. Sequencing was conducted using a paired-end, 2 × 251-bp cycle run on an Illumina MiSeq sequencing system and MiSeq Reagent Nano Kit version 2 (500 Cycle) chemistry. Paired-end sequencing with read lengths of about 251 bp was performed. After demultiplexing, a clear overlap in the pairedend reads was observed. This allowed paired reads to be joined together with the fastq-join program (http://code.google.com/p/ea-utils/). Only reads that that had quality value (QV) scores of ≥20 for more than 99 % of the sequence were extracted for further analysis. All sequences with ambiguous base calls were discarded [19].

### Bioinformatics analysis

The determined 16S rDNA sequences were subjected to homology searching using Metagenome@KIN software (World Fusion Co., Ltd., Tokyo, Japan) against the TechnoSuruga Lab Microbial Identification Databese DB-BA9.0 (TechnoSuruga Laboratory), which contains only bacteria with standing in the taxonomic nomenclature.

### Estimation of richness and diversity

Microbial diversity was assessed using the Shannon-Weiner diversity index (H’), which accounts for both the number of phylotypes (richness) and the proportion of the total accounted for by each phylotype (evenness) [22].

### Principal component analysis

Principal component analysis (PCA) was performed using Metagenome@KIN software (World Fusion Co., Ltd., Tokyo, Japan) based on data from bacterial genera with 97 % similarity cut-off with the Apollon DB-BA database, ver 9.0 (TechnoSuruga Laboratory).

### Statistical analysis

Data were analyzed using an unpaired *t*-test with Welch’s correction for continuous variables or the Mann-Whitney test (two-sided) and Fisher’s exact test for categorical variables using the IBM SPSS software Ver. 22. *P* values less than 0.05 were considered significant.

## Results

### Differences in bacterial community profiles between obese and non-obese subjects as determined by T-RFLP analysis

The characteristics of our subjects are shown in Table 1. A total of 23 non-obese subjects (BMI <20 kg/m^2^) and 33 obese subjects (BMI ≥25 kg/m^2^) were enrolled in this study. Blood test results showed that HbA1c, triglyceride, aspartate aminotransferase, and alanine aminotransferase levels were significantly higher and the high-density-lipoprotein cholesterol level was lower in the obese subjects. The average age of the obese subjects was higher than that of the non-obese subjects.

**Table 1:** Descriptive characteristics of study participants

	Non-obese (BMI <20 kg/m^2^)	Obese (BMI ≥25 kg/m^2^)	*P* value^b^
	n = 23	n = 33	
Age	45.6 ± 9.6^a^	54.4 ± 8.2	0.001
Gender, M; n (%)	11 (47.8)	20 (60.6)	0.417
BMI (kg/m^2^)	18.6 ± 1.2	27.8 ± 2.5	
Constipation; yes, n (%)	6 (26.1)	10 (30.3)	0.766
Alcohol intake; yes, n (%)	13 (56.5)	13 (39.4)	0.412
Smoking; yes, n (%)	5 (21.7)	7 (21.2)	1.000
Laboratory data			
HbA1c (JDS; %)	5.2 ± 0.4	5.7 ± 1.0	0.015
Total cholesterol (mg/dl)	192.7 ± 38.1	200.6 ± 52.4	0.464
Triglyceride (mg/dl)	78.4 ± 26.6	159.0 ± 108.1	<0.001
HDL-cholesterol (mg/dl)	78.4 ± 26.6	55.3 ± 14.4	0.001
AST (IU/l)	17.8 ± 6.5	23.7 ± 9.1	0.022
ALT (IU/l)	13.6 ± 5.8	33.4 ± 18.9	<0.001

*ALT* alanine aminotransferase, *AST* aspartate aminotransferase, *BMI* body mass index, *HDL* high-density lipoprotein, *JDS* Japan diabetes society

^a^Mean ± SD

^b^*P* values are based on two-sample *t*-test for continuous variables and Fisher’s exact test for categorical variables

Differences in bacterial flora between the two groups are summarized in Table 2. The relative proportion of *Bacteroidetes* at the phylum level was lower in stool samples obtained from obese subjects compared with non-obese subjects. The *Firmicutes* to *Bacteroidetes* ratio was higher in the stool samples obtained from obese subjects compared with non-obese subjects. There were no differences in other bacteria. There was no correlation between microbiota and subject age (Additional file 2: Table S2) and only HbA1c was weakly correlated with age in baseline variables (Additional file 3: Table S3).

**Table 2:** Differences in bacterial flora as determined by T-RFLP analysis

	Non-obese (BMI <20 kg/m^2^)	Obese (BMI ≥25 kg/m^2^)	*P* value^a^
*Actinobacteria* (Phylum)	8.2 ± 6.7 %^b^	8.0 ± 7.1 %	0.917
*Firmicutes* (Phylum)	37.0 ± 9.1 %	40.8 ± 15.0 %	0.241
*Bacteroidetes* (Phylum)	44.0 ± 9.8 %	37.0 ± 14.0 %	0.033
*Lactobacillales* (Order)	5.2 ± 9.2 %	7.2 ± 10.3 %	0.455
*Bifidobacterium*	8.2 ± 6.7 %	8.0 ± 7.1 %	0.917
*Bacteroides*	41.0 ± 11.8 %	35.1 ± 14.5 %	0.097
*Prevotella*	3.0 ± 7.3 %	2.0 ± 5.3 %	0.557
*Clostridium* cluster IV	8.0 ± 4.9 %	8.0 ± 6.2 %	1.000
*Clostridium* subcluster XIVa	21.7 ± 5.5 %	22.1 ± 9.5 %	0.841
*Clostridium* cluster XI	1.0 ± 1.4 %	1.7 ± 4.0 %	0.376
*Clostridium* cluster XVIII	1.3 ± 1.3 %	2.1 ± 2.0 %	0.080
*Firmicutes to Bacteroidetes* ratio	0.9 ± 0.4	1.7 ± 1.7	0.045

^a^*P* values are based on two-sample *t*-test with Welch correction

^b^Data are expressed as mean ± SD

### Differences in bacterial communities between obese and non-obese subjects as determined by 16S rRNA sequencing

**Fig. 2:**
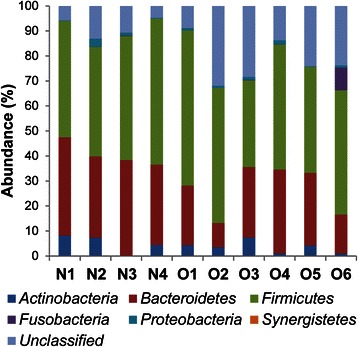
Phylum-level classification of bacteria identified in individual stool samples. N-numbered samples were obtained from non-obese subjects, whereas O-numbered samples were obtained from obese subjects. Each bar represents the percent contribution of phylum-level profiles grouped by non-obese-obese status or for each individual. The phyla represented by the different colors are shown below the figure

**Fig. 3:**
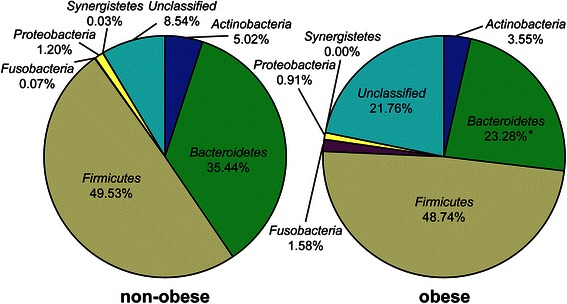
Average phylum distribution of gut microbiota of non-obese and obese patients. *P < 0.05

Our T-RFLP analysis revealed that the levels of *Bacteroidetes* would be significantly lower in obese subjects compared with non-obese subjects. To determine which *Bacteroidete* species differed in abundance, we selected samples from 10 subjects (4 non-obese and 6 obese) from the initial group of 55 for next-generation sequencing (Table 3). Using our primer set and MiSeq platform combination, an average of 39452 reads were obtained for each sequencing reaction. Figure 2 shows the phylotype distribution for individual patients in this study. The composition and relative abundance of the major phyla were similar, with *Bacteroidetes* and *Firmicutes* being the dominant phyla. However, after dividing the samples into two groups (obese vs. non-obese) and performing statistical analyses, a significant and drastic decrease in the proportion of *Bacteroidetes* (obese 23.28 % vs. non-obese 35.44 %; *P* < 0.05) and an increase in the proportion of “unclassified” phyla (obese 21.76 % vs. non-obese 8.54 %) were observed in the obese group relative to the non-obese group (Fig. 3). There were no differences in other bacteria.

**Table 3:** Characteristics of study participants whose gut microbiota was analyzed using next-generation sequencing

Participant ID	Health status	BMI (kg/m^2^)	Age	Gender, M : F
N1	non-obese	18.0	36.3 ± 4.1^a^	1 : 3
N2	non-obese	15.9		
N3	non-obese	17.9		
N4	non-obese	16.3		
O1	obese	27.4	51.7 ± 6.6^a^	5 : 1
O2	obese	25.8		
O3	obese	26.1		
O4	obese	26.3		
O5	obese	25.6		
O6	obese	32.8		

^a^Mean ± SD

### Differences in species diversity between obese and non-obese subjects

**Fig. 4:**
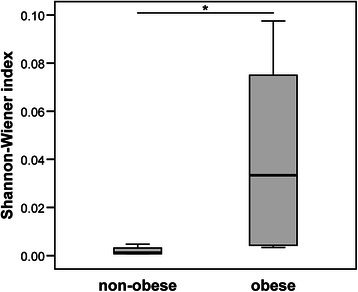
Comparison of bacterial diversity (Shannon-Wiener index) between the microbiota of non-obese and obese subjects. *P < 0.05

According to the phylotype classification at the genus level, we assessed microbial diversity based on measures of richness and evenness for non-obese and obese subjects using 16S rRNA gene sequences derived from clone libraries. The mean diversity results for non-obese and obese subjects are shown in Fig. 4. Microbial diversity and richness tended to be significantly higher in obese subjects compared with non-obese subjects (*P* < 0.05).

### Comparison of PCA results between obese and non-obese subjects

**Fig. 5:**
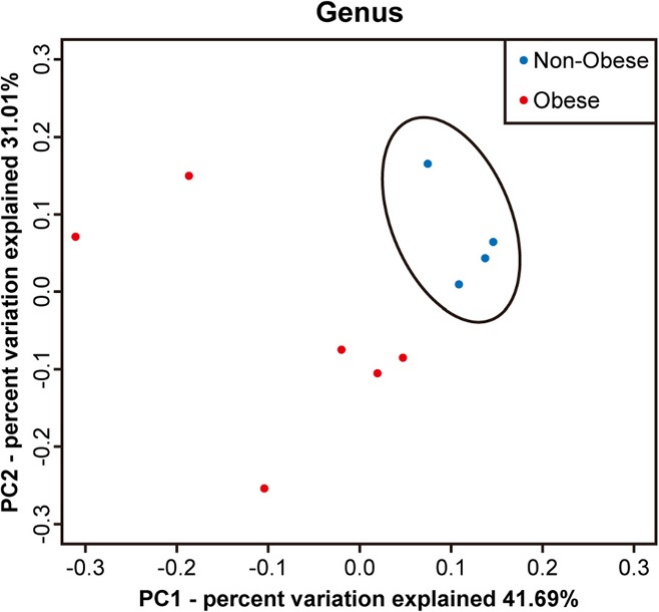
Comparison of principal component analysis results at the genus level between the gut microbiota of obese and non-obese subjects. A PCA based on dominant bacteria of PC1 (*Megamonas*, *Bacteroides*, and *Blautia*) and of PC2 (*Megamonas*, *Bacteroides*, and *Faecalibacterium*). Non-obese subjects formed a cluster (separated by a circle) distinct from obese subjects

PCA was performed based on dominant bacteria of PC1 (*Megamonas*, *Bacteroides*, and *Blautia*) and of PC2 (*Megamonas*, *Bacteroides*, and *Faecalibacterium*) at the genus level (Fig. 5). PCA results showed that 4 non-obese subjects formed a cluster (separated by a circle) distinct from obese subjects.

### Comparison of microbiomes at the species level

Species-level analyses identified five bacterial species that were significantly associated with the obese group: *Blautia hydrogenotorophica*, *Coprococcus catus*, *Eubacterium ventriosum*, *Ruminococcus bromii*, and *Ruminococcus obeum* (Table 4). It has been traditionally argued that the gut microbiota degrade and ferment resistant starches to produce short-chain fatty acids (SCFAs) that are used for energy harvesting by the host. The five above-mentioned species belong to the phylum *Firmicutes* and carry genes related to polysaccharide metabolism that enhance the efficiency of energy harvesting by the host.

**Table 4:** Bacterial species significantly more abundant in the stool of obese compared with non-obese individuals

	Ave. non-obese (%)	Ave. obese (%)	*P* value*
*Blautia hydrogenotorophica*	ND	0.01	0.040
*Coprococcus catus*	ND	0.21	0.030
*Eubacterium ventriosum*	ND	0.19	0.046
*Ruminococcus bromii*	ND	1.03	0.028
*Ruminococcus obeum*	0.07	0.87	0.038

*ND* not determined

*P values are based on Welch’s test

**Fig. 6:**
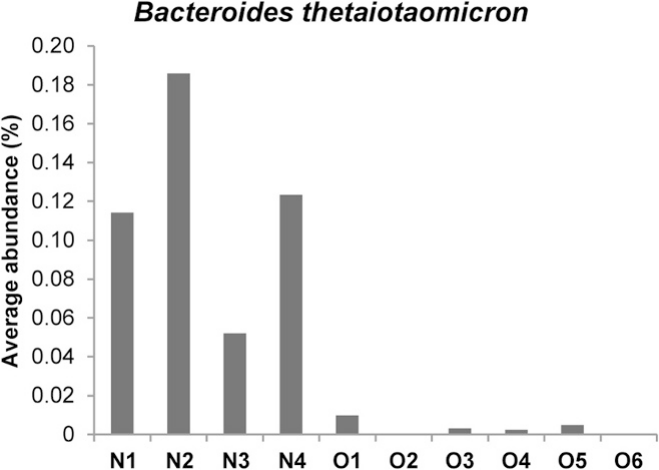
Average abundance of *Bacteroides thetaiotaomicron* in individual stool samples. N-numbered samples were obtained from non-obese subjects and O-numbered samples were obtained from obese subjects

**Fig. 7:**
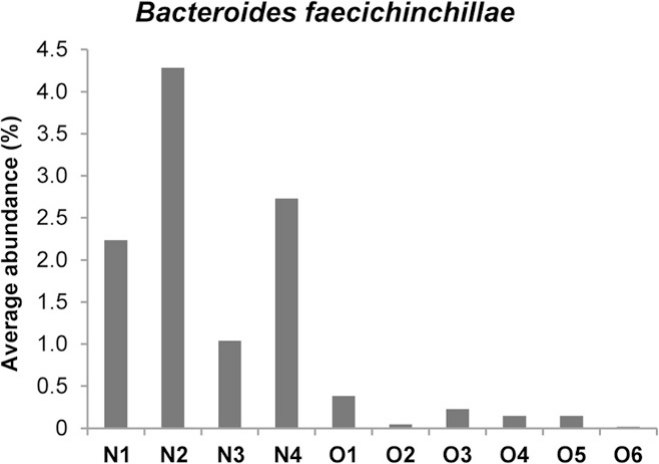
Average abundance of *Bacteroides faecichinchillae* in individual stool samples. N-numbered samples were obtained from non-obese subjects and O-numbered samples were obtained from obese subjects

Our results also showed that five bacterial species were significantly associated with the non-obese group: *Bacteroides faecichinchillae*, *Bacteroides thetaiotaomicron*, *Blautia wexlerae*, *Clostridium bolteae*, and *Flavonifractor plautii* (Table 5). Most notably, *Bacteroides faecichinchillae* and *Bacteroides thetaiotaomicron* were present in significantly greater proportions in the feces of non-obese subjects, whereas these bacteria were barely detectable in the feces of obese subjects (Figs. 6 and 7).

**Table 5:** Bacterial species significantly more abundant in the stool of non-obese compared with obese individuals

	Ave. non-obese (%)	Ave. obese (%)	*P* value*
*Bacteroides faecichinchillae*	2.57	0.16	0.037
*Bacteroides thetaiotaomicron*	0.12	ND	0.024
*Blautia wexlerae*	11.91	3.79	0.043
*Clostridium bolteae*	0.69	0.12	0.028
*Flavonifractor plautii*	0.22	0.06	0.038

*ND* not determined

*P values are based on Welch’s test

## Discussion

Using T-RFLP analysis and next-generation sequencing, we found that the composition of the gut microbiota differs between obese and non-obese subjects in a Japanese population. We obtained results similar to previous studies [10, 11] in terms of *Firmicutes* to *Bactericides* ratio but that were different from previous studies [12] in terms of diversity. We also identified potential bacterial species uniquely associated with each group. As generally proposed in previous studies [10, 11], T-RFLP analysis showed significantly reduced levels of *Bacteroidetes* and a higher *Firmicutes* to *Bactericides* ratio in obese subjects compared with non-obese subjects. In addition, the bacterial diversity of the gut microbiota was significantly greater in obese subjects compared with non-obese subjects in our study subjects.

Earlier studies revealed that the human gut microbiota becomes relatively stable around 1 week after birth, begins to resemble that of an adult after weaning, and once colonized in the gut of a healthy person remains stable over long period [23]. It is also traditionally thought that every healthy person has his or her own unique gut microbiota composition [2, 24, 25].

Past research investigating the relationship between the composition of the gut microbiota and the degree of obesity has yielded contradictory results. For example, it has been reported that an increase in *Firmicutes* and a decrease in *Bacteroidetes* is associated with obesity [10, 11, 26], whereas Schwiertz et al. reported a lower ratio of *Firmicutes* in overweight adults compared with lean controls [14], and Duncan et al. reported no differences between *Firmicutes* and *Bacteroidetes* according to BMI [15]. Although Duncan et al. hypothesized that the *Bacteroidetes* to *Firmicutes* ratio plays no important role in human obesity, at least at the phylum level, they did not rule out the possibility that discrepancies between the results of various researchers may be due to dietary habits and/or host physiology, as well as the methodologies used in the analyses.

Consistent with the reports of Ley, Furet, and Turnbaugh [10, 11, 26], our T-RFLP analyses in a Japanese population showed higher levels of *Bacteroidetes* and a lower *Firmicutes* to *Bacteroidetes* ratio at the phylum level in non-obese versus obese subjects. Furthermore, Mitsuoka et al. reported that modification of the human gut microbiota starts early in old age (at 65 years or over), although the cause is unclear [23]. It was for this reason that subjects over 65 years of age were excluded from our study. Although the average age of the obese subjects was higher than that of the non-obese subjects examined in the T-RFLP analysis in the current study, we found no correlations between any bacteria and subject age (Additional file 2: Table S2), indicating that the differences in gut microbiota between obese and non-obese subjects were not attributable to differences in the age of the subjects.

Following the T-RFLP analyses, we selected samples from several subjects from each group and analyzed them using next-generation sequencing followed by PCA. The results showed that the composition of the gut microbiota differs between obese and non-obese subjects, suggesting that changes in the gut microbiota composition are associated with body weight in the Japanese population we examined. In addition, compared with non-obese subjects, obese subjects exhibited greater gut bacterial diversity and richness. However, previous research conducted to date has shown that obesity is associated with reduced bacterial diversity [12, 27, 28]. Le Chatelier et al. studied the human gut microbial composition in 123 non-obese (BMI <25 kg/m^2^) and 169 obese (BMI >30 kg/m^2^) individuals in a Danish population and found that obese individuals with low bacterial richness were characterized by more noticeable overall adiposity, insulin resistance, dyslipidemia, and a more marked inflammatory phenotype when compared with obese individuals with a high gut bacterial richness [27]. Moreover, Cotillard et al. found that individuals with reduced microbial gene richness (40 %) presented with more pronounced dysmetabolism and low-grade inflammation than individuals with high bacterial gene richness [28]. Possible explanations for these discrepancies in bacterial diversity between previous studies and ours include the small number of study samples and differences in BMI categorization of the study subjects. In the literature from outside of Japan, lean and obese are defined as BMI <25 and >30 kg/m^2^, respectively, whereas in our study obese was defined as BMI ≥25 kg/m^2^ and non-obese as BMI < 20 kg/m^2^. Furthermore, in the analysis with next generation sequencing, only one patient had a BMI >30 kg/m^2^ and all of the non-obese subjects had a BMI ≤18 kg/m^2^ in this study. As the BMI categorization of our patient population differed from that of previous studies, it is difficult to directly compare the results. It is also possible that mildly obese persons have richer bacterial diversity, and that as obesity increases coupled with increasingly severe metabolic disturbances such as insulin resistance or dyslipidemia, the gut microbial diversity declines, which is consistent with the findings of Le Chatelier et al.

We found that five species, *Blautia hydrogenotorophica*, *Coprococcus catus*, *Eubacterium ventriosum*, *Ruminococcus bromii*, and *Ruminococcus obeum* (included in the *Firmicutes*), were significantly more abundant in stool samples obtained from obese subjects compared with non-obese subjects. The gut microbiota is involved in the fermentation of indigestible polysaccharides (components of dietary fibers) that are converted into SCFAs (e.g., acetate, propionate, butyrate) [29] used by the host as an energy source, representing 10-15 % of the energy influx from food [30]. All five of the species listed above are SCFA-producing bacteria belonging to the phylum *Firmicutes*, and it is likely that they provide energy to the host by promoting energy harvest and adipose tissue expansion [31]. However, Tagliabue et al. pointed out that this “energy harvest” hypothesis conflicts with epidemiologic data suggesting that high intake of dietary fiber (the main source of SCFAs) inhibits the development of obesity. That is to say, despite the recommendations for high dietary fiber intake as published by the World Health Organization and other groups as a means of enhancing weight loss or maintaining a healthier body weight, the energy harvest hypothesis suggests that high fiber intake leads to weight gain rather than weight loss. Thus, researchers have been studying mechanisms other than that associated with the energy harvest hypothesis [32]. In addition, Blaut et al. suggested that the gut microbiota may influence energy harvest by producing SCFAs from dietary fiber, but they also stressed that it is unknown whether this mechanism is relevant to human populations in Western countries, where the average intake of dietary fiber is quite low [33]. However, SCFA levels were not evaluated in our study, as this was not within the initial scope of our research; therefore, the relationship between obesity and energy harvest related to bacterial SCFA production should be investigated further.

In our study, populations of *Bacteroides faecichinchillae*, *Bacteroides thetaiotaomicron*, *Blautia wexlerae*, *Clostridium bolteae*, and *Flavonifractor plautii* were significantly more abundant in stool samples from non-obese compared with obese subjects. Most notably, two *Bacteroides* species (*B. faecichinchillae* and *B. thetaiotaomicron*) were detected in significant abundance in stool samples from all of the non-obese subjects but were barely detectable in samples from any of the obese subjects. According to Shipman et al., *Bacteroides thetaiotaomicron* is a gram-negative obligate anaerobe that can utilize polysaccharides very efficiently as a source of carbon and energy by binding them to the cell surface and allowing cell-associated enzymes to hydrolyze the polysaccharides into fragments that can be internalized by the bacterium [34]. This mechanism implies that this species independently harvests and consumes a certain amount of energy in the colon which would be otherwise used by the host. Furthermore, Ridaura et al. showed that co-housing obese and lean animals prevents the development of an increased adiposity phenotype as a result of invasion of the obese microbiota by specific *Bacteroidetes* within the lean microbiota. This microbial transition and the inhibition of body fat increase were observed only when animals were fed foods low in saturated fats and high in fruits and vegetables [35], suggesting that specific members of the gut microbiota contribute to the suppression of obesity and that the anti-obesity effect is diet dependent.

## Conclusion

In conclusion, the results of the current study in a Japanese population show that the gut microbiota differs between obese and non-obese individuals. However, whether alterations in the gut microbial composition are the cause or the sequel of obesity remains an open question for research. Further study will be necessary to elucidate the exact role of the gut microbiota in the development of lifestyle-related diseases such as obesity and diabetes.

## Additional files

Additional file 1: Table S1. Colonoscopy diagnosis of study participant. (DOCX 17 kb) Additional file 2: Table S2. Correlation between microbiota and patient age. (DOCX 20 kb) Additional file 3: Table S3. Correlation between baseline variables and patient age. (DOCX 19 kb)

## Abbreviations

BMI: Body mass index
T-RFLP: Terminal restriction fragment length polymorphism
SCFA: Short-chain fatty acid
