# Supplementary material for: Comparison of the gut microbiota composition between obese and non-obese individuals in a Japanese population, as analyzed by terminal restriction fragment length polymorphism and next-generation sequencing
Source: BMC Gastroenterol. 2015 Aug 11;15:100. doi: 10.1186/s12876-015-0330-2 (PMC4531509; doi:10.1186/s12876-015-0330-2)
Supplement: Additional file 1: Table S1. — Colonoscopy diagnosis of study participant. (DOCX 17 kb) [file 12876_2015_330_MOESM1_ESM.docx]

Supplementary Table 1. Colonoscopy diagnosis of study participant

|  | BMI<20 | BMI≥25 | Total |
| --- | --- | --- | --- |
| Normal findings | 14 | 7 | 21 |
| Nonspecific inflammation | 1 | 1 | 2 |
| Hyperplastic polyp | 1 | 2 | 3 |
| Adenoma | 6 | 20 | 26 |
| Carcinoma in adenoma | 1 | 1 | 2 |
| Early colon cancer | 0 | 2 | 2 |
| Total | 23 | 33 | 56 |
