# Supplementary material for: Comparison of the gut microbiota composition between obese and non-obese individuals in a Japanese population, as analyzed by terminal restriction fragment length polymorphism and next-generation sequencing
Source: BMC Gastroenterol. 2015 Aug 11;15:100. doi: 10.1186/s12876-015-0330-2 (PMC4531509; doi:10.1186/s12876-015-0330-2)
Supplement: Additional file 2: Table S2. — Correlation between microbiota and patient age. (DOCX 20 kb) [file 12876_2015_330_MOESM2_ESM.docx]

Supplementary Table 2. Correlation between microbiota and patient age

| ***Actinobacteria***  **(Phylum)** | r | -0.090 |
| --- | --- | --- |
|  | **P** | 0.509 |
| ***Firmicutes***  **(Phylum)** | r | 0.189 |
|  | **P** | 0.164 |
| ***Bacteroidetes***  **(Phylum)** | r | -0.199 |
|  | **P** | 0.142 |
| ***Lactobacillales***  **(Order)** | r | 0.224 |
|  | **P** | 0.097 |
| ***Bifidobacterium*** | r | -0.090 |
|  | **P** | 0.509 |
| ***Bacteroides*** | r | -0.169 |
|  | **P** | 0.213 |
| ***Prevotella*** | r | -0.043 |
|  | **P** | 0.754 |
| ***Clostridium* cluster IV** | r | 0.042 |
|  | **P** | 0.756 |
| ***Clostridium* subcluster**  **XIVa** | r | -0.004 |
|  | **P** | 0.974 |
| ***Clostridium* cluster XI** | r | -0.100 |
|  | **P** | 0.462 |
| ***Clostridium* cluster XVIII** | r | 0.196 |
|  | **P** | 0.148 |

**P* values and r values are based on Pearson correlation coefficient.
