# Supplementary material for: Comparison of the gut microbiota composition between obese and non-obese individuals in a Japanese population, as analyzed by terminal restriction fragment length polymorphism and next-generation sequencing
Source: BMC Gastroenterol. 2015 Aug 11;15:100. doi: 10.1186/s12876-015-0330-2 (PMC4531509; doi:10.1186/s12876-015-0330-2)
Supplement: Additional file 3: Table S3. — Correlation between baseline variables and patient age. (DOCX 19 kb) [file 12876_2015_330_MOESM3_ESM.docx]

Supplementary Table 3. Correlation between baseline variables and patient age

| **HbA1c**  **(JDS; %)** | r | 0.279 |
| --- | --- | --- |
|  | **P** | 0.045 |
| **TC** | r | 0.114 |
|  | **P** | 0.404 |
| **TG** | r | -0.246 |
|  | **P** | 0.739 |
| **HDL-C** | r | -0.005 |
|  | **P** | 0.972 |
| **ALT** | r | -0.047 |
|  | **P** | 0.775 |
| **AST** | r | 0.260 |
|  | **P** | 0.105 |

ALT, alanine aminotransferase; AST, aspartate aminotransferase; HDL-C, high-density lipoprotein-cholesterol; JDS, Japan diabetes society; TC, total cholesterol; TG, triglyceride

**P* values and r values are based on Pearson correlation coefficient.
